# A case report of TBL1XR1-RARB positive pediatric acute promyelocytic leukemia and literature review

**DOI:** 10.3389/fonc.2025.1566404

**Published:** 2025-08-29

**Authors:** Chunyan Du, Fu Liu, Linlin Huang, Ruxue Zeng, Jiamei Fan, Tao Hu

**Affiliations:** ^1^ Department of Pediatrics, Mianyang Central Hospital, School of Medicine, University of Electronic Science and Technology of China, Mianyang, China; ^2^ Pathological Diagnosis Center, Sichuan Kingmed Center for Clinical Laboratory Co., Ltd., Chengdu, Sichuan, China

**Keywords:** TBL1XR1-RARB fusion, atypical acute promyelocytic leukemia, clinical characteristics, immunophenotype, treatment

## Abstract

**Background:**

Acute promyelocytic leukemia (APL) is classically driven by the PML-RARA fusion oncogene and characterized by a maturation arrest of myeloid precursors. Variant APL (vAPL) with alternative RARA rearrangements presents diagnostic and therapeutic challenges.

**Methods:**

We report a novel case of TBL1XR1-RARB-positive vAPL and conducted a comprehensive literature review to synthesize clinical and molecular data from all previously reported cases of this rare entity.

**Results:**

Our patient presented with neutrophilic leukocytosis (15.44×10⁹/L) and an absence of peripheral promyelocytes, exhibiting fever as the sole symptom, an atypical CD45⁻/CD117⁻ immunophenotype, and a concurrent KRAS p.G12D mutation. Despite an initial response to ATRA/ATO therapy, relapse occurred during maintenance. Our literature review of all reported cases revealed key patterns: a predominant pediatric occurrence (median age 2.7 years), frequent ATRA/ATO resistance (55% response rate), and a high risk of relapse (44%).

**Conclusion:**

This study underscores the molecular heterogeneity and distinct clinical course of TBL1XR1-RARB-positive vAPL. It highlights significant therapeutic challenges, including a high rate of primary resistance and relapse, and provides critical guidance for the management of this rare but clinically significant disease.

## Introduction

Acute promyelocytic leukemia is a distinct form of acute myeloid leukemia (AML) (1). It is typically precipitated by the emergence of the PML-RARA fusion which is caused by the t(15;17)(q24;q21) translocation and which results in the synthesis of the PML-RARA fusion protein and the obstruction of the typical differentiation process of myeloid cells. It frequently exhibits a proclivity for severe bleeding tendency and may induce disseminated intravascular coagulation (DIC), which may result in early mortality. With the standardized use of all-trans-retinoic acid (ATRA) and arsenic trioxide (ATO), the long-term survival rate of APL patients can reach more than 95% (2–4). However, approximately 5% of APL patients exhibit typical morphological features but are negative for the PML-RARA fusion, referred to as variant APL (5, 6). Variant APL is primarily caused by RARA rearrangements other than PML-RARA and RARB or RARG rearrangements. Additionally, certain MLL rearrangements, NPM1 rearrangements, or other gene rearrangements can also lead to the development of variant APL (6). Recent studies have found that X::RAR::X or X::RAR::Y three-part fusion forms can also cause the development of variant APL (7).

The molecular pathogenesis of variant APL remains poorly understood. Unlike classical APL, variant APL demonstrates heterogeneous responses to ATRA and ATO, posing significant therapeutic challenges. Due to the absence of standardized treatment protocols and frequent intrinsic drug resistance, variant APL is associated with an unfavorable prognosis. Current clinical guidelines recommend ATRA combined with chemotherapy as first-line therapy for most variant APL cases. However, in instances of confirmed drug resistance, alternative AML-type chemotherapy regimens are typically employed (6). Among the rare genetic drivers of variant APL, TBL1XR1-RARB represents an exceptionally uncommon fusion. To date, the clinical characteristics, optimal treatment strategies, and long-term outcomes of TBL1XR1-RARB-positive APL have not been systematically investigated.

TBL1XR1 is a WD40 repeat-containing protein and a core component of the NCoR (nuclear receptor corepressor)/SMRT (silencing mediator of retinoic acid and thyroid hormone receptors) corepressor complex (8). This complex regulates transcriptional repression by interacting with nuclear hormone receptors (NRs) and specific transcription factors (9). TBL1XR1 functions primarily as an “exchange factor,” recruiting ubiquitin-conjugating enzymes and the 19S proteasome complex to mediate the ubiquitination and degradation of corepressors such as NCoR/SMRT and CtBP, thereby relieving repression and activating target gene expression (9).

We report a rare case of variant APL characterized by fever as the primary clinical manifestation, in the absence of typical bleeding tendencies or coagulation abnormalities. The patient tested positive for the TBL1XR1-RARB fusion gene and exhibited an atypical flow cytometric immunophenotype due to the absence of CD117 (a typical APL marker) and localization of leukemic cells in the CD45^-^ negative region, which is uncommon in classical APL. Despite receiving standard APL induction, consolidation, and re-consolidation therapy, the patient failed to achieve complete remission (CR). However, CR was successfully attained following the introduction of an AML-type consolidation regimen. Post-remission, the patient was maintained on a conventional high-risk APL therapy protocol. Unfortunately, relapse occurred during maintenance treatment, prompting consideration of hematopoietic stem cell transplantation (HSCT) as the next therapeutic step. Additionally, we conducted a comprehensive literature review to summarize the clinical features, genetic profiles, treatment responses, and prognostic outcomes of TBL1XR1-RARB-positive APL. This analysis aims to provide insights to guide future diagnosis and management of this rare and challenging disease variant.

## Case presentation

### Diagnostic evaluation

A previously healthy 1-year-and-11-month-old male presented to Mianyang Central Hospital on July 18, 2023, with a 15-day history of persistent high-grade fever (peaking at 39.5°C). The fever was unaccompanied by bleeding manifestations, significant anemia, or signs of extramedullary involvement. Despite out-of-hospital supportive treatment with antibiotics and intravenous human immunoglobulin (IVIG), the child continued to experience recurrent fever. Physical examination revealed an alert child with mild pharyngeal congestion but no cutaneous or mucosal hemorrhages. There was no hepatosplenomegaly or lymphadenopathy. The remainder of the physical examination was unremarkable, with normal cardiopulmonary and neurological findings. Cardiac evaluation showed right bundle branch block on ECG. Comprehensive imaging studies (cardiac/abdominal/testicular ultrasound, and CT of head/chest/abdomen) revealed no pathological findings. The child had no significant past medical history and his family history was negative for hematologic malignancies.

The patient’s pre-admission complete blood count (CBC) results from the referring hospital were as follows: Laboratory evaluation revealed leukocytosis (WBC 26.26×10^9^/L) with neutrophilia (15.44×10^9^/L), moderate anemia (Hb 85 g/L), and normal platelet count (240×10^9^/L). Inflammatory markers showed mild elevation (CRP 17.47 mg/L). Coagulation studies demonstrated hypofibrinogenemia (FIB 1.43 g/L) with elevated fibrinolytic markers (FDP 37.40 µg/mL, D-dimer 10.82 mg/L), while PT (11.9 s) and APTT (27.5 s) remained within normal limits. Peripheral blood smear examination showed minimal abnormal cells (1%) without identifiable promyelocytes. The patient’s CBC performed at our hospital on July 19, 2023 revealed leukocytosis (WBC 19.82×10^9^/L) with neutrophilia (10.98×10^9^/L), moderate anemia (Hb 81 g/L), and a normal platelet count (192×10^9^/L). Peripheral blood smear examination showed no evidence of abnormal cells or promyelocytes. The results of both tests are essentially consistent. Notably, this case presented with neutrophilic leukocytosis, which is atypical for classical APL characterized by maturation arrest.

**Figure 1:**
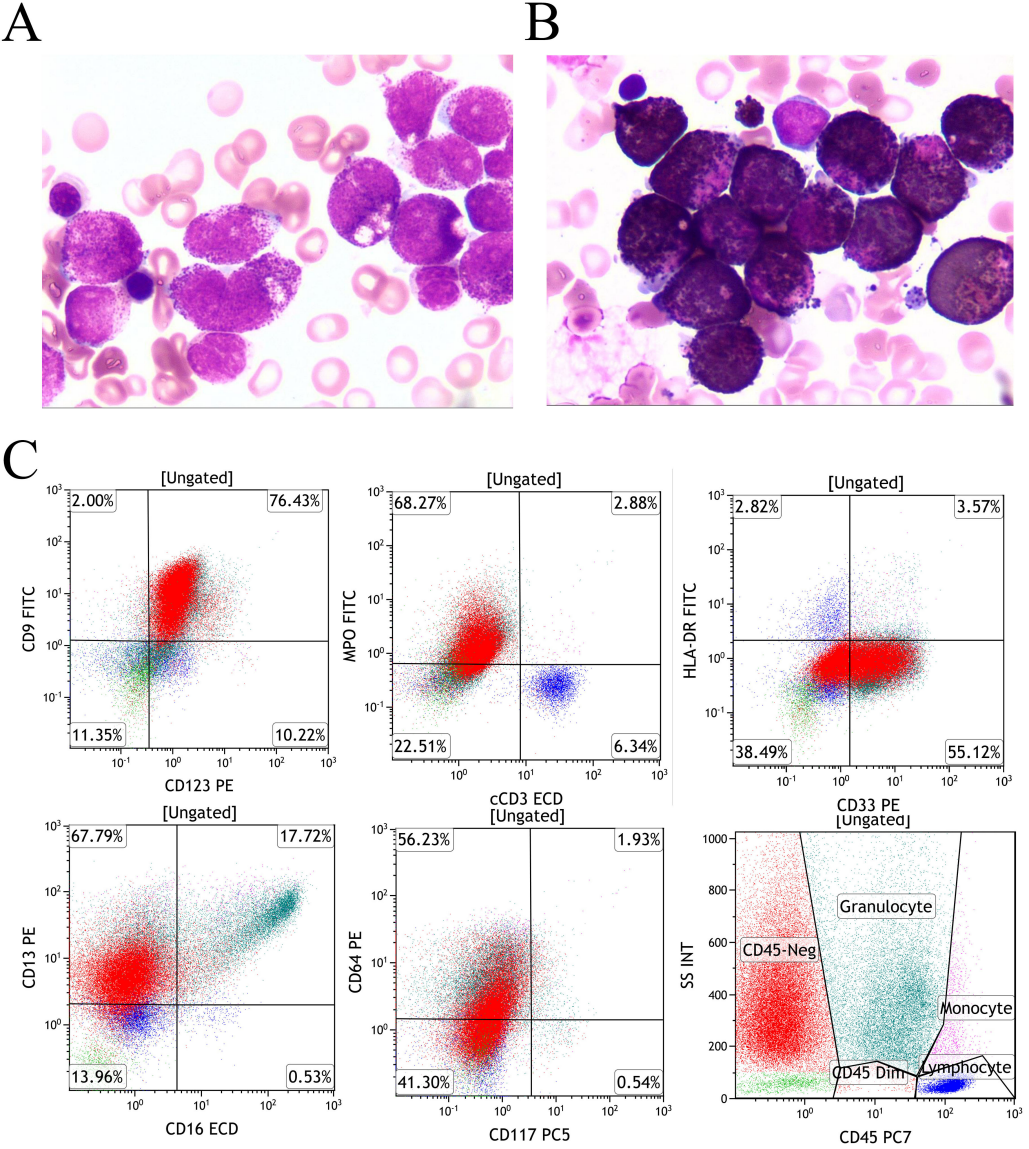
Morphology and immunophenotype **(A)** An increased number of promyelocytes, which contained large purplish-red granules (Wright-Giemsa staining of bone marrow, x1000); **(B)** Cytochemical demonstration of myeloperoxidase (MPO) activity using POX staining (Taiyang Biotechnology, China). Bone marrow smear showing intense POX positivity. **(C)** The leukemic population, residing in the C45-negative region, demonstrated positive expression of MPO, CD9, and CD123, with heterogeneous CD33, CD13, and CD64 expression, while remaining negative for HLA-DR and CD117.

Bone marrow evaluation demonstrated granulocytic hyperplasia (63.5% of ANC) with characteristic morphological features: abundant cytoplasm containing coarse purplish-red granules, showing strong MPO positivity (**Figures 1A, B**). Flow cytometry identified a predominant abnormal myeloid population (67.96% of nucleated cells) with a distinctive immunophenotype: positive for CD9, CD123, and MPO with heterogeneous CD33, CD13, and CD64 expression, while lacking CD45, CD117, CD34, HLA-DR, CD15, CD56, CD7, and CD2 expression (**Figure 1C**).

**Figure 2:**
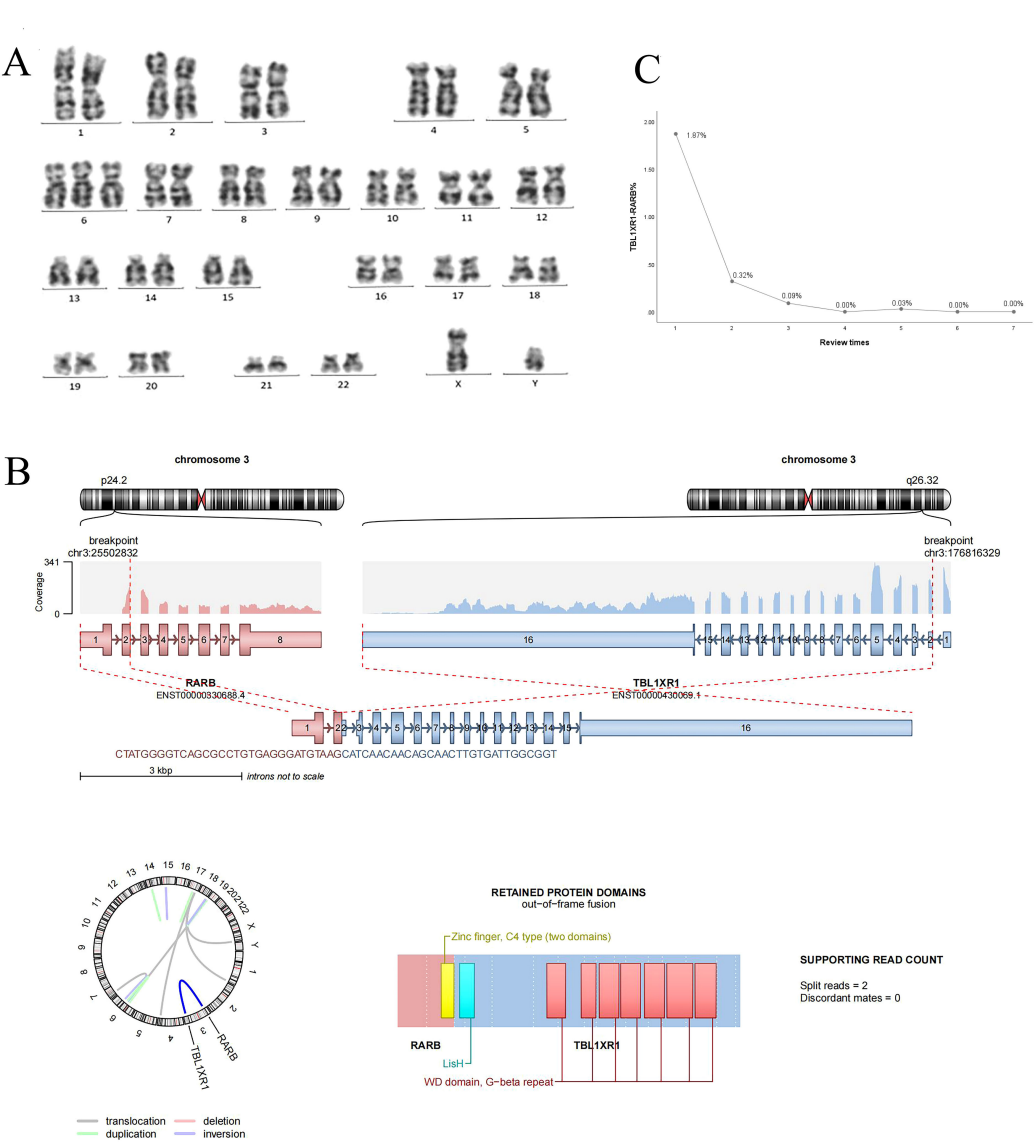
**(A)** Chromosome G band analysis revealing karyotype 46,XY,+6 [20]; **(B)** TBL1XR1-RARB fusion gene; **(C)** TBLIXRI-RARB fusion gene detected after induction, after consolidation l, after consolidation 2, after consolidation 3, before maintenance, after maintenance l and 2, respectively.

At the time of initial diagnosis, the child’s bone marrow was evaluated through cytomorphometry and flow cytometric immunophenotyping, revealing an atypical immunophenotype consistent with acute promyelocytic leukemia (APL) (CD34^-^/HLA-DR^-^/CD45^-^CD117). Cytogenetic analysis via G-banded karyotyping, performed in accordance with the International System for Human Cytogenomic Nomenclature (2020) (10), showed no evidence of the classic t(15;17) translocation. Cytogenetic analysis revealed a karyotype of 47, XY, +6[20] (**Figure 2A**). Initial screening for PML-RARA fusion by RT-PCR was negative, prompting further molecular investigation.

To identify the underlying genetic driver, whole transcriptome RNA sequencing (RNA-seq) was performed using the Illumina NovaSeq 6000 platform. Data analysis included: 1) Fusion detection using Arriba (GRCh37 reference); 2) Variant calling with VarScan (minimum VAF 1%); 3) Pathogenicity classification per AMP/ASCO/CAP tiers. All Tier-1 variants were confirmed by orthogonal methods. We identified a clinically significant (Tier-1) *TBL1XR1-RARB* fusion with 17 supporting reads (**Figure 2B**), classified as pathogenic based on AMP/ASCO/CAP guidelines. Concurrently, a KRAS p.G12D hotspot mutation was detected at 24.3% variant allele frequency, a known activating mutation in AML. Additionally, 12 variants of uncertain significance (VUS) were identified, including SPEN p.P2574L (56.6% VAF) and POT1 p.F565L (43.5% VAF), though their clinical relevance in APL remains unclear. No other pathogenic fusion genes were detected. Droplet digital PCR (ddPCR) was subsequently employed to confirmed the presence of *TBL1XR1-RARB* fusion.

### Treatment course

**Figure 3:**
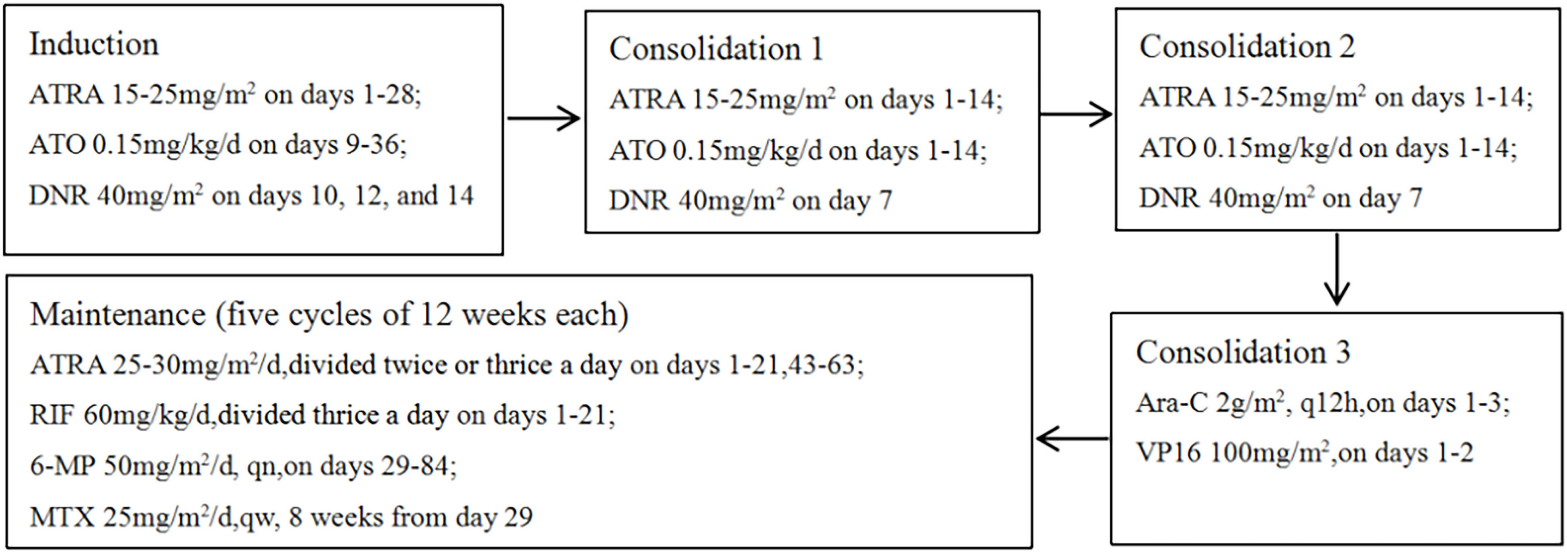
Treatment regimens. ATO, arsenic trioxide; ATRA, all-trans retinoic acid; DNR, daunorubicin; Ara-C, cytarabine; Vp16, etoposide; RlF, realgar-indigonaturalis; 6-MMP, 6-mercaptopurine; MTX, methotrexate.

The patient’s initial diagnosis of acute promyelocytic leukemia was established based on characteristic bone marrow morphological features and immunophenotypic profile. On July 18, 2023, treatment was initiated with a high-risk APL induction regimen consisting of ATRA, ATO, and daunorubicin, aiming to induce differentiation and apoptosis of leukemic cells. Subsequent molecular testing on July 21, 2023 revealed negative results for the PML-RARA fusion transcript, raising strong suspicion for variant APL. Confirmatory testing through comprehensive fusion analysis identified the rare TBL1XR1-RARB fusion transcript. Notably, despite existing literature suggesting resistance of TBL1XR1-RARB-positive APL to differentiating agents, our patient demonstrated a favorable clinical response to ATRA and ATO, manifested by gradual defervescence and the development of differentiation syndrome, which was successfully managed with dexamethasone. In light of this positive response, we continued treatment with the standard high-risk APL protocol. Supportive care included piperacillin-tazobactam for infection prophylaxis and hydroxyurea for leukocytosis management. Post-induction evaluation revealed complete morphological remission with negative minimal residual disease by flow cytometry. However, sensitive molecular monitoring detected persistent TBL1XR1-RARB fusion transcripts at 1.87% by digital PCR (**Figure 2C**), a level comparable to high-risk thresholds in classical APL (11). The patient subsequently underwent two cycles of consolidation therapy with ATO, ATRA, and daunorubicin, maintaining morphological and immunophenotypic remission with progressively decreasing but persistently detectable fusion transcript levels. Given the suboptimal molecular response and reported resistance patterns in variant APL, treatment was intensified with an AML-type consolidation regimen incorporating cytarabine and etoposide. This approach successfully achieved complete molecular remission with undetectable fusion transcripts. The observed response to initial ATRA/ATO therapy, while unexpected based on existing literature, suggested potential heterogeneity in treatment sensitivity among TBL1XR1-RARB-positive cases. Following achievement of complete remission, maintenance therapy was initiated using the standard high-risk APL protocol combining ATRA, realgar-indigo naturalis formula, 6-mercaptopurine, and methotrexate. Unfortunately, the patient experienced disease recurrence during maintenance therapy, prompting consideration of allogeneic hematopoietic stem cell transplantation as the next therapeutic approach. The complete treatment timeline is detailed in **Figure 3**.

### Review of the literature

A comprehensive review of published literature through November 2024 identified six case reports documenting nine pediatric patients with TBL1XR1-RARB-positive variant APL. Borkovskaia et al. (2023) identified TBL1XR1-RARB and KMT2A-SEPT6 fusions in RARA-negative pediatric AML with APL-like morphology, noting resistance to ATRA/ATO and advocating for RNA-seq to improve diagnostic accuracy (12). Zhao et al. (2019) expanded this spectrum, revealing RARB/RARG fusions (e.g., TBL1XR1-RARB, CPSF6-RARG) and KMT2A rearrangements in morphologically similar cases, with RARA-negative patients showing inferior survival and higher relapse rates despite AML-style chemotherapy (13). Osumi et al. (2018) characterized TBL1XR1-RARB as a recurrent driver in RARA-negative APL, demonstrating its dominant-negative suppression of RAR signaling and clinical resistance to retinoids (14). These findings were corroborated by a 2021 case report of a 2-year-old boy with TBL1XR1-RARB APL, where initial ATRA/RIF therapy achieved transient remission, but subsequent AML-style chemotherapy (FLAG-IDA) and targeted maintenance ensured durable remission (15). Collectively, these studies underscore that RARB/RARG-rearranged and KMT2A-fused APL-like leukemias represent distinct entities with unique molecular drivers, necessitating early genetic profiling to guide therapy—typically AML regimens for RARA-negative cases—while highlighting TBL1XR1-RARB as a biomarker for ATRA resistance and potential HSCT candidacy. Standard immunophenotyping (CD13+/CD33+/HLA-DR+) cannot differentiate TBL1XR1-RARB from PML-RARA APL, underscoring the need for routine cytogenetic/molecular profiling in pediatric AML with APL-like features.

The cohort comprised five male and four female patients, with ages ranging from 11 months to 4.8 years. Clinical presentation data revealed that one-third of cases (3/9) presented with fever as a prominent symptom, while only one patient manifested concurrent bleeding tendencies and anemia at diagnosis. Hematologic parameters at presentation showed significant variability: leukocyte counts ranged from 6.1 to 128.3×10^9^/L (median 32.4×10^9^/L), with all patients demonstrating anemia. Thrombocytopenia was nearly universal, with only one case maintaining normal platelet levels. Morphologically, all cases fulfilled diagnostic criteria for acute promyelocytic leukemia (M3 morphology), confirmed by characteristic AML immunophenotypic profiles (**Table 1**). Therapeutic responses exhibited notable heterogeneity. Differentiation therapy outcomes showed that 55% (5/9) of patients responded to ATRA, while only 22% (2/9) demonstrated sensitivity to ATO, albeit with reduced efficacy compared to classical APL. All cases maintained chemosensitivity to conventional agents. Treatment outcomes revealed that 44% (4/9) of patients underwent hematopoietic stem cell transplantation (HSCT) at various disease stages. Clinical courses included one induction failure, four relapses (44%), and one mortality following multiple relapses. One patient was lost to follow-up, while the remaining seven patients (78%) achieved sustained remission (**Table 2**). These findings highlight both the clinical variability and therapeutic challenges associated with this rare APL variant.

**Table 1:** Clinical features and laboratory tests.

Reference	No.	Age (years)	Gender	Clinical future	WBC (×10^9^/L)	PLT (×10^9/^L)	HB (g/L)	Morphology	Immunophenotype	Cytogenetics	Mutations
Osumi (14)	1	2.6	M	NA	53.11	NA	NA	FAB-M3	CD13^+^CD33^+^HLA-DR^+^CD34^-^	46,XY,t(3;10;12)(q26.2;q22;q15)[20/20]	NA
	2	4.3	F	NA	6.1	NA	NA	FAB-M3	CD13^+^CD33^+^HLA-DR^+^CD34^+^	46,XX,del(2)(p)?,inv(4)(p16q12)[1/20],45,idem,-X[3/20],46,idem,del(3)(p25)[3/20],46,XX[13/2]	NA
	3	4.1	F	NA	14.9	NA	NA	FAB-M3	CD13^+^CD33^+^HLA-DR^+^CD34^+^	47,XX,+3 [19/20]	NA
Zhao (13)	4	0.98	M	NA	128.3	34	98	FAB-M3	NA	46,XY	ATXN3,MAP2K2
	5	4.76	M	NA	24.3	54	84	FAB-M3	NA	46,XY	STAG2
Shiba (16)	6	0.9	F	NA	30.5	NA	NA	FAB-M3	NA	47,XX,16[2]/46,XX	NA
Jiang (17)	7	2	F	Fever,ecchymosis,anemia	41.0	29	74	FAB-M3	CD13^+^CD15^+^CD33^+^CD64^+^ MPO^+^	46,XX	EZH2, TBL1XR1
Zhang (15)	8	2.8	M	Fever	24.82	105	72	FAB-M3	NA	46,XY	KRAS,NRAS, TBL1XR1
Borkovskaia (12)	9	1	F	NA	60	NA	NA	FAB-M3	CD33+ CD13+ CD15+ CD99+ MPO+	46,XX	NA
This study	10	1.9	M	Fever	26.6	240	85	FAB-M3	CD9^+^CD123^+^CD33^+^CD64^+^CD13^+^MPO^+^	47,XY,+6[20]	KRAS

NA, not applicable.

**Table 2:** Treatment response and prognosis.

Reference	No.	Response to ATRA	Response To ATO	Chemotherapy	HSCT	Events	Time to relapse from diagnosis	Outcome
Osumi (14)	1	None	NA	Yes	Yes	relapse	6 months	Alive
	2	None	NA	Yes	Yes	Induction failure	NA	Alive
	3	None	NA	Yes	Yes	relapse	13 months	Alive
Zhao (13)	4	Yes	NA	Yes	None	None	None	Alive
	5	Yes	NA	Yes	None	relapse	29.7 months	Death
Shiba (16)	6	NA	NA	Yes	Yes	relapse	NA	Alive
Jiang (17)	7	Yes	Yes	Yes	None	None	NA	Alive
Zhang (15)	8	Yes	Yes	Yes	None	None	NA	Alive
Borkovskaia (12)	9	Yes	NA	Yes	None	NA	NA	NA
This study	10	Yes	Yes	Yes	None	relapse	15 months	Alive

ATRA, all-trans retinoic acid; ATO, arsenic trioxid; HSCT, Hematopoietic Stem Cell Transplantation, NA, not applicable.

## Discussion

### Consistency with previous studies

Classical PML-RARA-positive APL typically manifests with significant coagulopathy, characterized by severe bleeding diathesis and frequent development of DIC, which accounts for substantial early mortality in untreated cases (15, 17). In striking contrast, the TBL1XR1-RARB variant demonstrates a distinct clinical phenotype, typically presenting without coagulation abnormalities or overt hemorrhagic manifestations. Notably, approximately 11% of pediatric cases with this variant maintain normal platelet counts throughout their clinical course. The most common and frequently sole presenting symptom in TBL1XR1-RARB-positive APL is persistent fever, which serves as the primary clinical indicator in the majority of cases. In our case, fever was the primary symptom, while hemorrhagic manifestations—typical of classical APL—were absent. Current literature indicates that TBL1XR1-RARB-positive APL affects preschool-aged children, with reported cases ranging from 0.9 to 4.76 years (median 2.7 years) (13–17). Our patient’s age at onset (1.9 years) falls within this characteristic early-onset pattern. The suboptimal response to ATRA/ATO and eventual relapse mirror the resistance patterns observed in reported cases, underscoring the need for alternative strategies such as AML-type chemotherapy or HSCT (6, 13, 14).

### Unique features of this case

Morphologically, our case demonstrated the classic M3 features typical of TBL1XR1-RARB-positive APL. The immunophenotypic profile showed some deviations from conventional APL patterns: while most APL cases exhibit strong CD117 expression (a well-established diagnostic marker), our patient surprisingly demonstrated CD117 negativity. This finding carries prognostic significance, as CD117-negative APL has been associated with poorer outcomes, including higher early mortality rates and reduced overall and progression-free survival (18, 19). The immunophenotype also showed absence of CD56, CD15, and CD2 - markers typically associated with adverse outcomes when expressed. CD56 positivity in particular has been correlated with increased risk of extramedullary involvement and treatment resistance (20–22). The CD45^-^/CD117^-^profile contrasts with classical APL (typically CD45^+^/CD117^+^) and may reflect origin from primitive progenitors, which not described in prior literature. This unique “CD45^-^CD117^-^” immunophenotype is exceptionally rare in clinical practice, suggesting the leukemic cells may have originated from more primitive hematopoietic progenitor cells than conventional APL.

KRAS mutations predominantly occur at critical amino acid residues (G12, G13, Q61, and A146) and are frequently implicated in various hematologic malignancies, including acute lymphoblastic leukemia (ALL), myelodysplastic syndromes (MDS), juvenile myelomonocytic leukemia (JMML), and AML (23). In this case, RNA sequencing of the patient’s bone marrow identified a KRAS p.G12D missense mutation—a well-characterized hotspot variant that constitutively activates RAS protein signaling. This gain-of-function mutation promotes sustained downstream pathway activation, contributing to dysregulated proliferation and survival of leukemic cells. The KRAS p.G12D variant, though not previously linked to TBL1XR1-RARB APL, aligns with its role in myeloid malignancies (13). Its functional impact warrants further study.

In contrast to classical APML, our patient presented with marked leukocytosis (26.26×10^9^/L) and neutrophilia (ANC 15.44×10^9^/L) with only rare abnormal cells identified on peripheral smear review. This atypical presentation may reflect either: 1) the presence of concurrent infection may have contributed to both the neutrophilic response and the obscuration of abnormal promyelocytes in peripheral blood smears, or 2) fundamental biological differences in the peripheral blood manifestations of TBL1XR1-RARB positive APML compared to classical PML-RARA positive disease, or 3) the typical APL cases are expected to exhibit maturation arrest; therefore, this case could potentially represent an extremely rare form of APL without maturation arrest. Multiple manual peripheral smear reviews confirmed the paucity of circulating abnormal promyelocytes (abnormal cell percentage 0.0%), supporting that this was not simply an artifact of automated analysis. This unusual hematologic presentation expands the known spectrum of variant APML manifestations and underscores the importance of molecular diagnostics when clinical and laboratory findings appear discordant with classical disease features.

Unlike most reported TBL1XR1-RARB cases with ATRA resistance (13, 14), our patient initially responded to ATRA/ATO, suggesting biologic heterogeneity. However, relapse during maintenance highlights the limitations of differentiation therapy alone.

### Clinical implications

The RARB gene is located at chromosome 3p24, while TBL1XR1 resides at 3q26.32. The TBL1XR1-RARB fusion gene typically arises from t(3;3) or inv(3) rearrangements. However, unlike some other APL variants, TBL1XR1-RARB-positive cases have not been associated with loss of heterozygosity on chromosome 3, making this fusion particularly difficult to detect via conventional karyotyping. In our patient, cytogenetic analysis revealed a gain of chromosome 6 (+6) without structural abnormalities of chromosome 3, consistent with prior reports of this rare fusion. Structurally, RARB shares approximately 90% homology with RARA and RARG, all of which belong to the retinoic acid receptor (RAR) family and exhibit overlapping transcriptional regulatory functions. TBL1XR1 encodes an F-box/WD40 repeat-containing protein that serves as a critical component of the nuclear receptor corepressor complexes NCoR and SMRT. These complexes mediate transcriptional repression by unliganded NRs and other transcription factors (TFs) (24). Notably, TBL1XR1 can form homodimers that interfere with both RARA and RARB signaling. To date, TBL1XR1 is the only identified molecular chaperone for RARB. The TBL1XR1-RARB fusion oncoprotein mimics the leukemogenic mechanisms of PML-RARA by dysregulating the retinoic acid receptor pathway and blocking myeloid differentiation (11). Despite this mechanistic similarity, TBL1XR1-RARB-positive APL demonstrates intrinsic resistance to standard differentiating agents, including ATRA, ATO, and the synthetic retinoid tamibarotene (6, 11, 14, 25).

The therapeutic efficacy of ATRA in classical APL stems from its direct binding to the RARA moiety of PML-RARA, inducing conformational changes that facilitate co-repressor complex dissociation and co-activator recruitment, thereby reactivating the RARA transcriptional network. Concurrently, ATRA promotes PML-RARA degradation through the ubiquitin-proteasome system (26, 27). ATO exerts its effects by targeting specific cysteine residues in the zinc finger domains of PML, inducing sumoylation and subsequent proteasomal degradation of PML-RARA (28–30). The synergistic interaction between ATRA and ATO has been well-documented in clinical trials, contributing to the remarkable 95% long-term survival rate in APL, though approximately 5% of patients experience refractory or relapsed disease, particularly those with high-risk features or variant APL (3, 4). Patients harboring the TBL1XR1-RARB fusion typically demonstrate reduced sensitivity to ATRA/ATO therapy compared to classical APL (6). Our patient achieved morphological remission (negative bone marrow cytology and MRD) following induction therapy, yet persistent minimal residual disease was evident by quantitative detection of TBL1XR1-RARB (1.87%). While no established threshold exists for TBL1XR1-RARB in variant APL due to its rarity, insights can be drawn from PML-RARA monitoring in classical APL. Studies using ddPCR have demonstrated that MRD levels >0.1% post-consolidation correlate with increased relapse risk in PML-RARA-positive APL (11). Furthermore, pretreatment PML-RARA transcript levels >209.6 copies/ng by ddPCR independently predict relapse (31). In our case, persistent TBL1XR1-RARB at 1.87% post-induction prompted therapy escalation, supporting the hypothesis that low-level residual disease (even <1%) may be clinically significant. Given ddPCR’s superior sensitivity (LOD: 2.9 copies/μL vs. qPCR’s 98 copies/μL) (32), we propose: TBL1XR1-RARB levels >0.1% (ddPCR) should trigger closer surveillance. Transcripts >1% post-consolidation may warrant treatment intensification (e.g., HSCT). Further studies are needed to validate these thresholds in TBL1XR1-RARB-positive APL.

Subsequent consolidation therapy resulted in progressively declining fusion transcript levels, suggesting that while the TBL1XR1-RARB-positive leukemia retained some responsiveness to ATRA/ATO, this sensitivity was markedly diminished compared to classical APL. The cumulative daunorubicin dose of 200 mg/m² approached established cardiotoxicity thresholds, necessitating alternative therapeutic approaches. Current evidence supports the use of high-dose cytarabine consolidation for high-risk APL (33, 34) and AML-type regimens for treatment-resistant variant APL cases (34–38). All reported cases of TBL1XR1-RARB-positive APL have been managed with ATRA/ATO combined with chemotherapy, with three patients ultimately undergoing hematopoietic stem cell transplantation. Our patient achieved molecular remission following intensive consolidation incorporating high-dose cytarabine and etoposide. The observed gradual decline in fusion transcript levels during initial ATRA/ATO/daunorubicin therapy suggests some degree of preserved sensitivity to differentiating agents, albeit significantly reduced compared to classical APL. Given the substantial toxicity profile associated with intensive AML-type regimens, particularly the elevated risk of infectious complications, maintenance therapy with ATRA, RIF, 6-MP, and MTX was implemented. The emergence of disease relapse during maintenance therapy prompted consideration of hematopoietic stem cell transplantation as a definitive treatment approach. Notably, the treatment course was not complicated by severe infections or cardiotoxicity. While the combination of ATRA/ATO with chemotherapy may represent a viable initial approach for TBL1XR1-RARB-positive APL, the observed high relapse rate underscores the potential necessity of hematopoietic stem cell transplantation as a curative intervention for this variant form of APL.

## Conclusion

We report a novel case of TBL1XR1-RARB-positive vAPL with fever as the sole symptom, atypical CD45^-^/CD117^-^ immunophenotype, and KRAS p.G12D mutation. This case expands the phenotypic spectrum of TBL1XR1-RARB-positive vAPL by demonstrating neutrophilic leukocytosis —features suggestive of a maturation arrest-deficient subtype. Our case and literature review highlight the need for early molecular diagnosis, intensified chemotherapy, and consideration of HSCT due to high relapse risk. The absence of standardized MRD monitoring thresholds for this fusion underscores the urgent need for molecularly guided treatment protocols. Future efforts should focus on establishing international registries to facilitate multicenter collaborative studies, which will be critical for defining evidence-based diagnostic and therapeutic guidelines, elucidating the molecular mechanisms underlying treatment resistance and developing targeted approaches for this rare but clinically significant APL variant.

## Data Availability

The datasets presented in this study can be found in online repositories. The names of the repository/repositories and accession number(s) can be found in the article/supplementary material.
